# Trigeminal Neuralgia: A Clinical Review for the General Physician

**DOI:** 10.7759/cureus.3750

**Published:** 2018-12-18

**Authors:** Muhammad H Majeed, Sadaf Arooj, Muhammad Abbas Khokhar, Tamoor Mirza, Ali A Ali, Zahid H Bajwa

**Affiliations:** 1 Psychiatry, Natchaug Hospital, East Lyme, USA; 2 Radiology, Holy Family Hospital, Rawalpindi, PAK; 3 Oncology, King Edward Medical University, Lahore , PAK; 4 Psychiatry, Royal Darwin Hospital, Darwin, AUS; 5 Psychiatry, Icahn School of Medicine at Mount Sinai, Elmhurst Hospital Center, Flushing, USA; 6 Pain Medicine, Tufts University School of Medicine, Boston, USA

**Keywords:** trigeminal neuralgia, tic douloureux, headaches, pain management, neurology, general psychiatry, primary care

## Abstract

General practitioners (GPs) are often the first clinicians to encounter patients with trigeminal neuralgia (TN). Given the gravity of the debilitating pain associated with TN, it is important for these clinicians to learn how to accurately diagnose and manage this illness. The objective of this article is to provide an up-to-date literature review regarding the presentation, classification, diagnosis, and the treatment of TN. This article also focuses on the long-term management of these patients under the care of GPs. GPs play an important role in the management of patients with TN by following the evidence-based management guidelines. The most important aspects of the management of TN are discussed in this review article.

## Introduction and background

Trigeminal neuralgia (TN) is also called tic douloureux. The first account of TN is found in the writing of Avicenna in the 11^th^ century, but it was John Fothergill who gave the modern description of TN in his 1773 paper on the subject [1]. In the 21^st^ century medicine, the pathophysiology of TN has been established through a deeper understanding of the physiology and development of imaging techniques. Due to its episodic nature, its initial presentation and the long-term management of these patients fall under the care of the general practitioners (GPs). 

## Review

Prevalence

A survey of the GPs in the United Kingdom reported the incidence of TN as 26 per 100,000 per year, between 1992 to 2002 [2]. The National Institute of Neurological Disorders and Stroke estimates the incidence rate of 12 per 100,000 people per year [3]. There is gender variability in the incidence of TN with a female to male ratio of 1.74:1 with most of the cases occurring after 50 years of age [4].

Presentation

**Figure 1:**
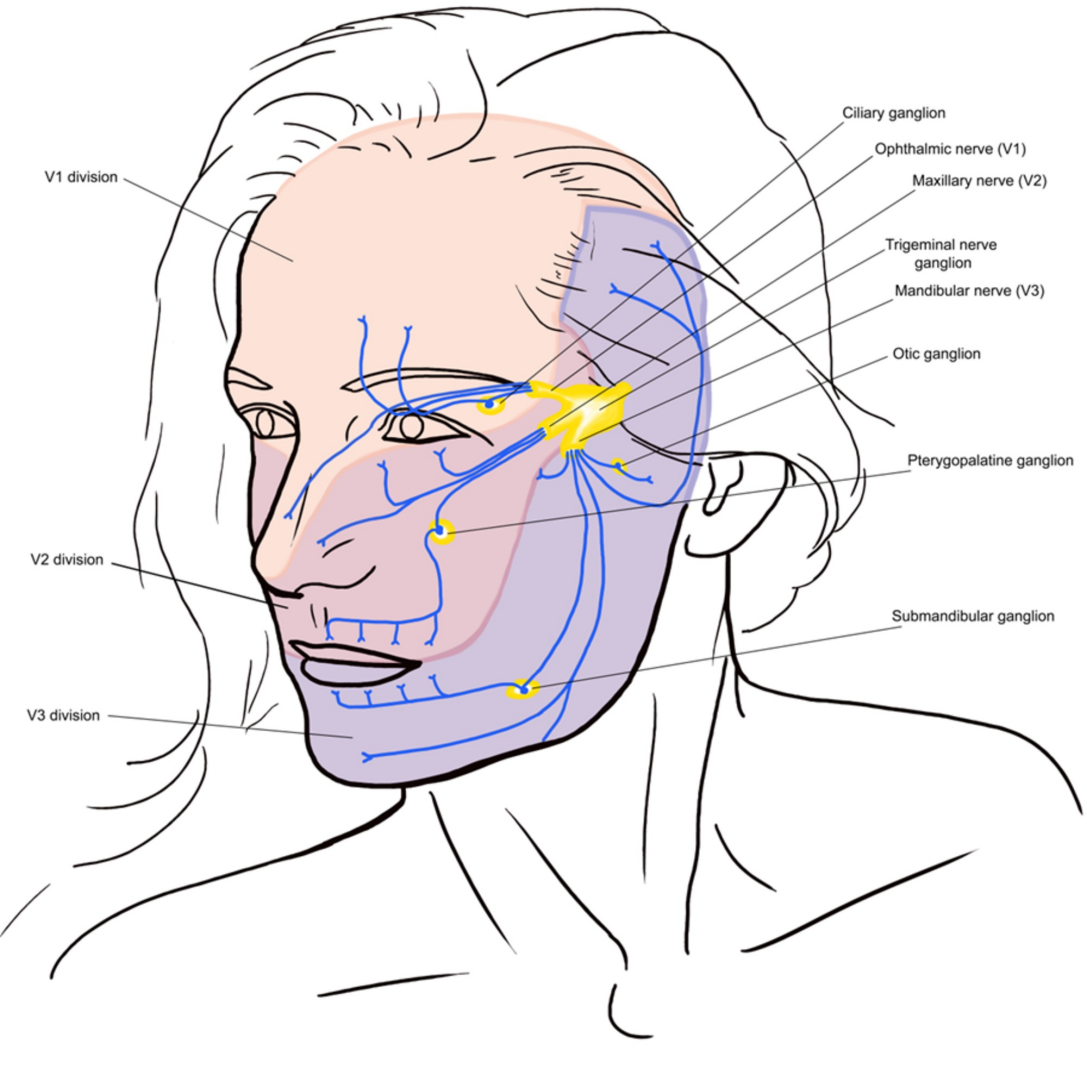
Overview of distribution of the trigeminal nerve and its terminal branches

Almost 87% of all facial pains are related to dental or oral mucosal lesions [5]. The most common causes of facial and jaw pain are listed in Table 1. Clinical history, examination, investigations, and imaging are important to make a correct diagnosis [6]. TN is characterized by an abrupt onset and short-lived unilateral shock-like pain, limited to the distribution of the trigeminal nerve. Triggers for classical TN (CTN) usually include mastication (73%), touch (69%), tooth brushing (66%), eating (59%), talking (58%), and cold wind on the face (50%). Combing hair is not usually a common trigger for TN. There can be concomitant background pain within the distribution of the nerve. Trigger zones are present in more than 90% of the patients, with touch and vibrations being the most common stimuli in provoking pain. Pain is usually distributed along the V2 and V3 branches (Figure 1). Pain occurs slightly more often (59% to 66%) on the right side of the face and rarely (3% to 5%) is bilateral. Its diagnostic criteria from The International Classification of Headache Disorders (ICHD-3) are summarized in Table 2.

**Table 1:** Common causes of facial pain

Common Causes of Facial Pain
Oral cavity and salivary gland lesions (infection, trauma, inflammation, space-occupying lesion)
Facial bones and joint diseases
Paranasal sinus disease
Neuro-vascular disorders
Psychosomatic disorders

**Table 2:** ICHD-3 diagnostic criteria for trigeminal neuralgia ICHD-3: International Classification of Headache Disorders

ICHD-3 Diagnostic Criteria for Trigeminal Neuralgia
A. Pain has all of the following characteristics:
1. Lasting from a fraction of a second to two minutes
2. Severe intensity
3. Electric shock-like, shooting, stabbing or sharp in quality
B. Precipitated by innocuous stimuli within the affected trigeminal distribution
C. Not better accounted for by another ICHD-3 diagnosis

Clinicians should be aware of other disorders such as painful trigeminal neuropathy that can present like TN. Herpes Zoster infestation of the trigeminal ganglion of the ophthalmic nerve, chronic paroxysmal hemicrania, Tolosa-Hunt syndrome, migraine, cluster headache, and glossopharyngeal neuralgia are among the differential diagnoses of TN. A list of differential diagnoses is presented in Table 3.

**Table 3:** Differential diagnosis of trigeminal neuralgia HZ: Herpes Zoster, TN: trigeminal neuralgia, SUNHA: short-lasting unilateral neuralgiform headache attacks

Diagnosis	Location	Frequency	Nature	Demographic	Associated symptoms	Treatment
Glossopharyngeal neuralgia	Unilateral; ear, tonsils, larynx, and tongue	Paroxysmal	Electrical or lancinating	Slightly more common in women	Triggered by swallowing or coughing; spontaneous remissions	Pharmacologic treatment is similar to TN.
Cluster headache	Unilateral; ocular, frontal, and temporal areas	Episodic; 15 to 180-minute episodes	Stabbing, burning, and throbbing	18-40 years Males	Ipsilateral; ptosis, miosis, tearing, and rhinorrhea	High-flow 100% oxygen; ergot preparations; prednisone; methysergide
Chronic paroxysmal hemicrania	Ocular, frontal, and temporal areas	Multiple five to 45-minute episodes	Stabbing, burning, and throbbing	Females	Ipsilateral conjunctival injection, lacrimation	Indomethacin
SUNHA	Unilateral; periorbital, neuralgiform headache	15 to 120-second episodes	Burning, stabbing, or electric pain	23 to 77 years; males	Conjunctival injection, tearing, rhinorrhea, and facial flushing	Corticosteroids; anti-epileptic drugs
Tolosa-Hunt syndrome	Unilateral; retro-orbital	Constant	Steady gnawing or boring	20 years and above; males and females	Ophthalmoplegia; spontaneous resolution	Corticosteroids
HZ involving the trigeminal ganglion	Unilateral; ophthalmic division in the majority of cases	Constant	Burning pain, sometimes accompanied by neuralgic pain	60 to 70 years; males and females	Vesicular eruption within seven days	Antivirals; steroids

Classification

In the third edition of the ICHD-3, TN is sub-classified into classical, secondary, and idiopathic causes [7]. In CTN, pain occurs along the distribution of TN without any obvious reasons other than neurovascular compression. CTN has recurrent paroxysms of unilateral facial pain and involves pain-free periods or concomitant background facial pain. Persistent background pain, bilateral symptoms, the patient’s age less than 50 years, focal neurological signs, and sensory impairment raise the suspicion for etiologies other than CTN. Multiple sclerosis (MS), space-occupying lesions, and neuropathy are among the common causes of secondary TN (STN). STN is caused by underlining pathology, and frequently on clinical examination, sensory abnormalities could be elicited. Patients experience unilateral facial pain in paroxysmal fashion and may have background continuous or near continuous pain. Multiple sclerotic plaques at the trigeminal root entry zone or in the pons, causing impairment in the TN pathway, are the most common cause of STN. About 2% of patients with MS have TN [8]. A space-occupying lesion coming in contact with TN can cause recurrent paroxysms of unilateral facial pain. Idiopathic TN is a condition that shows the classical symptoms of TN, but neither electrophysiological tests nor radiological investigations show significant abnormalities. In this review article, we will primarily discuss CTN.

Etiology and pathophysiology

Several observations lead to the vascular-compression theory of CTN, which indicates that TN is caused by the pressure of blood vessels on the trigeminal nerve as it exits the brain stem. Most commonly, a rostroventral superior cerebellar artery loop compresses the trigeminal nerve and causes the symptoms [9]. 

Diagnosis

CTN is a clinical diagnosis based on the history of the patient and a thorough physical exam, particularly a neurological exam. Magnetic resonance imagining/angiography (MRI/MRA) is often used to confirm the diagnosis and to exclude other possible causes of facial pain. Imaging techniques can help locate the area of the neurovascular loop as well as find any secondary causes. Neurophysiological recording of trigeminal brainstem reflexes and trigeminal evoked potentials help detect the lesion [7]. Medical treatment for most patients with CTN is required. Medical therapy helps provide relief from excruciating pain and decrease in pain frequency and duration as well as the associated symptoms. Patients who are resistant to or unable to tolerate medications can be candidates for surgical treatment.

Treatment

The European Federation of Neurological Societies and the Quality Standards Subcommittee of the American Academy of Neurology consider carbamazepine (CBZ) as the drug of choice for the treatment of TN [10]. CBZ is also the only Food and Drug Administration (FDA)-approved medication for the treatment of TN. CBZ is an anticonvulsant that is structurally close to tricyclic compound imipramine. It works by the inhibition of sodium channel activity and the modulation of calcium channels. Its efficacy in the treatment of TN is well established. In a review of several studies, CBZ provided a high level of pain control (58% to 100%), while the placebo success rate was only 0% to 40% [10]. However, providers should be aware of tachyphylaxis with the use of CBZ. The typical starting dose is 100 to 200 mg twice daily and then is gradually increased to 200 mg. The usual maintenance dose is 600 to 1200 mg in divided doses with a desired therapeutic blood level of 4 to 12 ug/ml. The initial half-life of CBZ is around 30 hours, but because of auto-induction of cytochrome P450 isoenzyme CYP3A4, it is reduced to 10 to 12 hours. This decrease in half-life necessitates monitoring the serum levels, twice daily dose scheduling, or use of a time-release formulary. The presence of the human leukocyte antigen (HLA)-B*15:02 allele is a genetic susceptibility marker of developing Stevens-Johnson syndrome in high-risk (Asians) populations. Patients with Asian ancestry should be screened for this allele before starting CBZ, and if positive, the use of CBZ should be avoided. More common side effects include drowsiness, dizziness, and nausea. Severe side effects include aplastic anemia, hyponatremia, and abnormal liver function tests. Regular monitoring of blood counts, liver function tests, and serum sodium levels is recommended.

Oxcarbazepine (OXC) is an analog of CBZ that has better tolerability, predictable metabolism, and fewer interactions with other medications. Both OXC and CBZ were proven equally effective in the reduction of pain attacks and intensity in at least three head-to-head trials [10]. Serum sodium levels should be periodically monitored in patients taking OXC. Some small, open-label studies support the use of pregabalin, gabapentin, topiramate, levetiracetam, and valproic acids as well [11]

Local anesthetics such as alcohol, glycerol, phenol, tetracaine, or bupivacaine injections are used in the diagnosis and treatment of TN [12]. These percutaneous injections can provide relief from pain for a few months to years. Injections of botulin toxin A also help reduce the pain intensity and frequency in TN [13]. Patients, whose symptoms are refractory to medical treatment or if they are unable to tolerate it due to side effects, are preferred candidates for surgical procedures for the treatment of TN. Usually, surgical therapies involve decompression of the nerve by resetting the vascular loop around the nerve or sometimes resetting the nerve that carries the pain signals [14].

Percutaneous trigeminal ganglion balloon compression rhizotomy is usually reserved for patients who cannot tolerate the above-mentioned treatments or are refractory to it [15]. Percutaneous radiofrequency gangliolysis, trigeminal ganglion compression, and retrogasserian glycerol rhizolysis are different techniques utilized to ablate the ganglion. These procedures have high initial success rates. Microvascular decompression (MVD) is conducted under general anesthesia using a microscope to identify the trigeminal nerves. The artery or vein causing pressure on the trigeminal nerve is removed or repositioned to relieve the compression. This procedure is performed endoscopically as well [16]. Stereotactic radiosurgery with Gamma-knife or Cyber-knife radiosurgery is also used in the treatment of TN [17-18]. Peripheral neurectomy is reserved for patients who have failed medical and other conservative surgical treatments or are not suitable candidates for them [19]. The development of atypical facial pain is a common after-effect of the procedure. TN is usually chronic and may take a psychological toll on the patient as well. The National Institute of Neurological Disorders and Stroke, a subdivision of the National Institute of Health, United States, provides information, treatment, and support to patients suffering from TN and their families [3]. To check for the efficacy of treatment or its side effects, patients should be monitored at regular intervals with their general physicians. The recurrence of symptoms is common after medical or surgical treatment.

## Conclusions

TN is a rare disease but often associated with debilitating pain and disability. Various neurological and infectious causes can mimic its symptoms, and the exact diagnosis is recommended prior to initiation of treatment. CBZ is the drug of choice and the only FDA medication approved for TN. Other treatment options include neuroleptic blocks and surgical alternatives. Given the insidious nature of the disease, general physicians play a crucial role in the management of TN.

## References

[1] Pearce JMS (2003). Trigeminal neuralgia (Fothergill’s disease) in the 17th and 18th centuries. J Neurol Neurosurg Psychiatry.

[2] Hall GC, Carroll D, Parry D (2006). Epidemiology and treatment of neuropathic pain: the UK primary care perspective. Pain.

[3] National Institute of Neurological Disorders and (2018). National Institute of Neurological Disorders and Stroke. Trigeminal neuralgia fact sheet. Trigemincal Neuralgia Fact Sheet. https://www.ninds.nih.gov/Disorders/Patient-Caregiver-Education/Fact-Sheets/Trigeminal-Neuralgia-Fact-Sheet. Accessed.

[4] Katusic S, Beard CM, Bergstralth E, Kurland Kurland, LT LT (1990). Incidence and clinical features of trigeminal neuralgia, Rochester, Minnesota, 1945-1984. Ann Neurol.

[5] Stovner LJ, Hagen K, Jensen R (2007). The global burden of headache: a documentation of headache prevalence and disability worldwide. Cephalalgia.

[6] Quail G (2005). Atypical facial pain--a diagnostic challenge. Aust Fam Physician.

[7] Headache Classification Committee of the International Headache Society (IHS) (2013). The International Classification of Headache Disorders, 3rd edition (beta version). Cephalalgia.

[8] Hooge JP, Redekop WK (1995). Trigeminal neuralgia in multiple sclerosis. Neurology.

[9] Love S, Coakham HB (2001). Trigeminal neuralgia pathology and pathogenesis. Brain.

[10] Cruccu G, Gronseth G, Alksne J (2008). AAN-EFNS guidelines on trigeminal neuralgia management. Eur J Neurol.

[11] Gronseth G, Cruccu G, Alksne J (2008). Practice parameter: the diagnostic evaluation and treatment of trigeminal neuralgia (an evidence-based review): report of the Quality Standards Subcommittee of the American Academy of Neurology and the European Federation of Neurological Societies. Neurology.

[12] Goto F, Ishizaki K, Yoshikawa D, Obata H, Arii H, Terada M (1999). The long lasting effects of peripheral nerve blocks for trigeminal neuralgia using high concentration of tetracaine dissolved in bupivacaine. Pain.

[13] Zhang H, Lian Y, Ma Y, Chen Y, He C, Xie N, Wu C (2014). Two doses of botulinum toxin type A for the treatment of trigeminal neuralgia: observation of therapeutic effect from a randomized, double-blind, placebo-controlled trial. J Headache Pain.

[14] Parmar M, Sharma N, Modgill V, Naidu P (2013). Comparative evaluation of surgical procedures for trigeminal neuralgia. J Maxillofac Oral Surg.

[15] Kanpolat Y, Savas A, Bekar A, Berk C (2001). Percutaneous controlled radiofrequency trigeminal rhizotomy for the treatment of idiopathic trigeminal neuralgia: 25-year experience with 1,600 patients. Neurosurgery.

[16] Bohman L, Pierce J, Stephen JH, Sandhu S, Lee JK (2014). Fully endoscopic microvascular decompression for trigeminal neuralgia: technique review and early outcomes. Neurosurg Focus.

[17] Baschnagel AM, Cartier JL, Dreyer J (2014). Trigeminal neuralgia pain relief after gamma knife stereotactic radiosurgery. Clin Neurol Neurosurg.

[18] Karam SD, Tai A, Snider JW (2014). Refractory trigeminal neuralgia treatment outcomes following CyberKnife radiosurgery. Radiat Oncol.

[19] Ali F, Prasant M, Pai D, Aher AV, Kar S, Safiya T (2012). Peripheral neurectomies: a treatment option for trigeminal neuralgia in rural practice. J Neurosci Rural Pract.

